# Concanavalin A as a promising lectin-based anti-cancer agent: the molecular mechanisms and therapeutic potential

**DOI:** 10.1186/s12964-022-00972-7

**Published:** 2022-10-26

**Authors:** Huldani Huldani, Ahmed Ibraheem Rashid, Khikmatulla Negmatovich Turaev, Maria Jade Catalan Opulencia, Walid Kamal Abdelbasset, Dmitry Olegovich Bokov, Yasser Fakri Mustafa, Moaed E. Al-Gazally, Ali Thaeer Hammid, Mustafa M. Kadhim, Seyed Hossein Ahmadi

**Affiliations:** 1 Department of Physiology, Faculty of Medicine, Lambung Mangkurat University, Banjarmasin, South Kalimantan Indonesia; 2 Department of Pharmacology, Collage of Medicine, University of Babylon, Hilla, Iraq; 3 Department of Clinical Pharmacology, Samarkand State Medical Institute, Samarkand, Uzbekistan; 4 Department of Scientific Affairs, Tashkent State Dental Institute, Makhtumkuli Street 103, Tashkent, Uzbekistan 100047; 5 College of Business Administration, Ajman University, Ajman, UAE; 6 Department of Health and Rehabilitation Sciences, College of Applied Medical Sciences, Prince Sattam Bin Abdulaziz University, Al Kharj, Saudi Arabia; 7 Department of Physical Therapy, Kasr Al-Aini Hospital, Cairo University, Giza, Egypt; 8 Institute of Pharmacy, Sechenov First Moscow State Medical University, 8 Trubetskaya St., Bldg. 2, Moscow, 119991 Russian Federation; 9 Laboratory of Food Chemistry, Federal Research Center of Nutrition, Biotechnology and Food Safety, 2/14 Ustyinsky Pr, Moscow, 109240 Russian Federation; 10 Department of Pharmaceutical Chemistry, College of Pharmacy, University of Mosul, Mosul-41001, Iraq; 11 College of Medicine, University of Al-Ameed, Karbala, Iraq; 12 Computer Engineering Techniques Department, Faculty of Information Technology, Imam Ja’afar Al-Sadiq University, Baghdad, Iraq; 13 Department of Dentistry, Kut University College, Kut, Wasit 52001 Iraq; 14 College of Technical Engineering, The Islamic University, Najaf, Iraq; 15 Department of Pharmacy, Osol Aldeen University College, Baghdad, Iraq; 16 Research Center for Cell and Molecular Sciences, School of Medicine, Tehran University of Medical Sciences, PO Box 1417613151, Tehran, Iran

**Keywords:** Lectin, Concanavalin A, Anti-neoplastic agent, Autophagy, Apoptosis, Cancer

## Abstract

Concanavalin A (ConA), the most studied plant lectin, has been known as a potent anti-neoplastic agent for a long time. Since initial reports on its capacity to kill cancer cells, much attention has been devoted to unveiling the lectin's exact molecular mechanism. It has been revealed that ConA can bind to several receptors on cancerous and normal cells and modulate the related signaling cascades. The most studied host receptor for ConA is MT1-MMP, responsible for most of the lectin's modulations, ranging from activating immune cells to killing tumor cells. In this study, in addition to studying the effect of ConA on signaling and immune cell function, we will focus on the most up-to-date advancements that unraveled the molecular mechanisms by which ConA can induce autophagy and apoptosis in various cancer cell types, where it has been found that P73 and JAK/STAT3 are the leading players. Moreover, we further discuss the main signaling molecules causing liver injury as the most significant side effect of the lectin injection. Altogether, these findings may shed light on the complex signaling pathways controlling the diverse responses created via ConA treatment, thereby modulating these complex networks to create more potent lectin-based cancer therapy.

**Video Abstract**

**Supplementary Information:**

The online version contains supplementary material available at 10.1186/s12964-022-00972-7.

## Introduction

Advancements in cancer medicine have caused a descendence either in cancer occurrence or its mortality. However, it is still expected that the number of people suffering malignancies will rise exponentially in the next few decades. Therefore, there will be an increasing desire to find new agents that address both diagnostic and therapeutic goals. As bioactive proteins, lectins are a central hub in this domain. Lectins are non-immune origin proteins possessing binding affinity toward glycoconjugates in a specific and reversible manner. In cancer biology, Lectins in the past have been exploited as the biological sensors detecting the degree of glycosylation in cancerous cells to distinguish between malignant and benign tumors [1–5]. The legume family of plant lectins is one of the most investigated classes because of their significant biological functions, and the best-representing lectin of this potent family is Concanavalin A (ConA).

**Fig. 1:**
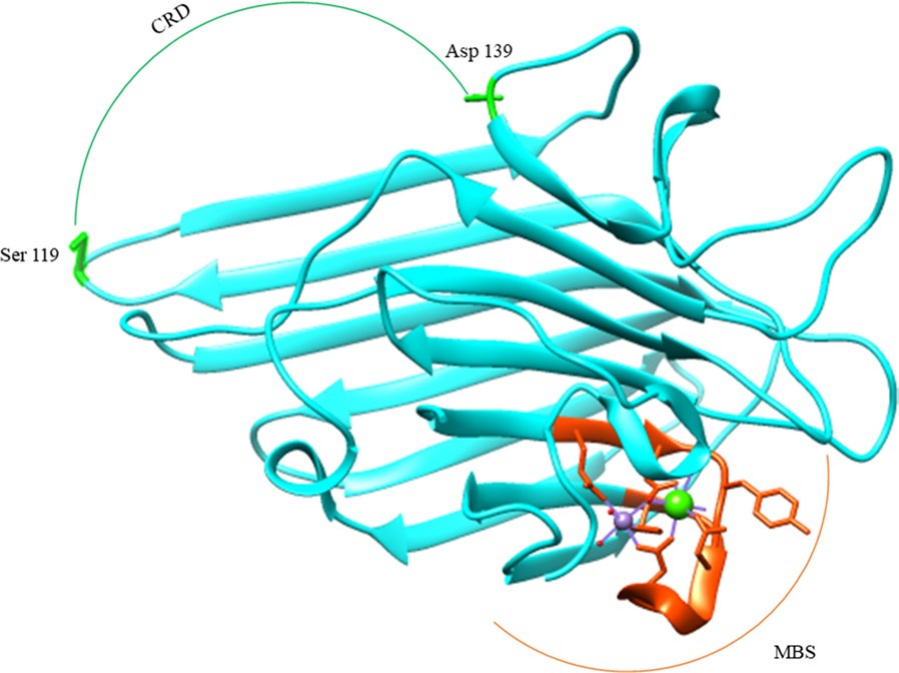
The 3-D structure of the native form of ConA monomer (1NLS) presents the jellyroll motif. ConA is the foremost characterized leguminous lectin as it is the first isolated lectin. Its monomer has a molecular rate of ~ 25 KDa, with 237 residues, arranging a canonical 12-stranded β-sandwich structure. Of note, the oligomerization status of the lectin depends on the pH values. The green circle indicates the carbohydrate recognition domain (CRD) in which the involved residues are illustrated as sticks; the orange circle shows the metal binding site (MBS) interacting with two metal ions, Ca^2+^ (in green) and Mn^2+^ (in purple), that play a pivotal role in lectin stability

At first, over 100 years ago, ConA deciphered as a defense glycoprotein from Jack bean Cannavalia Ensiformis. After that, ConA became the first legume lectin sequenced and crystallized, which revealed a monomeric 273 residue glycoprotein (Mw = 27KDa) with a secondary structure composed of β-sandwich strands that need metal ions for its suitable folding, stability, and function (Fig. 1). Also, α-D-Man, α-D-Glu, and β-D-Fru were among the most interactional moieties ConA tends toward. ConA, in terms of physiochemical properties, is classified as a stable lectin withstanding a broad range of pH (5–8) and temperatures (up to 60 °C) without loss of function. The environmental pH is the primary factor determining the oligomerization state of the lectin, where at the pH of 5.8–7, ConA has its biological native tetravalent form. In contrast, the pH < 5.8 and > 7 have lower and higher valencies, respectively [6–9].

ConA is a renowned lectin with outstanding mitogenicity, and similar to other plant lectins, it is naturally toxic. The glycan-binding capacity of lectins determines their mitogenicity, which relies on the lectin affinity for carbohydrates located on immune cell receptors. The lectin shows diverse biological functions based on its different sugar-binding affinity to distinct cell types. It discriminates the normal and cancerous cells in vitro and prevents tumor establishment in vivo. In high concentrations, > 20 mg/g, it arouses autophagy on hepatoma cells (hepatocyte cytotoxic), while in low concentrations, 1–10 mg/g, it activates lymphocytes (mitogenic). ConA can bind to several cellular receptors in different cell types, leading to their clustering and tyrosine phosphorylation, then altering the activity of dozens of downstream effectors. Inducing autophagy and apoptosis, activating immune and stem cells, and inhibiting/inducing angiogenesis are the most studied functions of the most studied lectin in plants, ConA [10–18]. Outstanding anti-tumor functions alongside the cytotoxic manifestations have been appealing growing attempts toward developing modifications that mitigate ConA cytotoxicity. PEG modification, for example, potently declined its cytotoxicity. In addition, other approaches like site-directed mutagenesis of the lectin are developing, which enables generating a safer lectin for clinical implications without losing its competence [8, 9, 11]. Furthermore, little is known regarding the bioavailability of ConA which should be further investigated in future studies. This paper takes a more profound look at the signaling pathways modulated by different concentrations of ConA in different cell types and/or contexts. Elucidation of the whole molecular mechanisms controlling various cellular responses to ConA may open up a new perspective for its possible future clinical usage. If proven clinically safe, this could open novel avenues in lectin-based cancer medicine.

## ConA receptors

There are several cell surface receptors reported as the host receptors for ConA. However, the central signal transducer and the most studied receptor of ConA biology is MT1-MMP (Membrane-Type Matrix MetalloProteinase-1). It evidenced that the tumor cells benefit from MT1-MMPs since they cause a simpler metastasis and neovascularization by activating different MMPs [19–21]. MMPs are the zn^2+^-dependent proteases typically organized into three distinct classes: collagenases (MMP-1), gelatinases (MMP-2), and stromelysins (MMP-3). Most MMPs become activated through the cleavage by soluble MMPs or serine proteases. Conversely, the precursor of MMP-2, pro-MMP-2, is only susceptible to MT1-MMP [21–25]. The helical spots of collagen type- IV of the basement membrane are the substrate of MMP-2; that is why it is also named 72 KDa type- IV collagenase [26]. MMP-2 secretes as a pro-enzyme in a complex with its inhibitor, tissue inhibitor of metalloproteinase-2 (TIMP-2). Forming a three-member complex is required to recognize pro-MMP2 by MT1-MMP receptors. In this process, the N-terminus domain of TIMP-2 binds to the catalytic domain of MT1-MMP, while its C-terminus domain binds to its counterpart on the pro-MMP2 protein [24, 27]. In addition to MMPs activation, MT1-MMP, in parallel, can stimulate a protease storm through activating the matripase serine proteases [28]. Matripase is a member of the s1 trypsin-like serine proteases, which mediates ECM degradation and angiogenesis by activating several downstream factors such as HGF and uPA. Moreover, Matripase has an autoprocessing activity causing its degradation; so preventing this process entails forming a complex of a second protein; HAI-1 (hepatocyte growth factor activator inhibitor-1) plays this role as TIMP-2 performs in inhibiting MMP-2 autocatalytic activity. Matripase-HAI-1 complex accumulates in the cytoplasm and upon activation, the processed form of matripase translocates on the cell surface [28]. Furthermore, two other mechanisms by which MT1-MMP augments tumor progression are the shedding of the adhesion molecule CD44 and the cleavage of MHC-1 chain-related molecule A from the tumor cell surface, which makes them resistant to killing by NK (Natural Killer) cells. Hence, MT1-MMP as a sensor and an effector might act as a switch to decide whether ECM degradation occurs [29].

PZR (protein zero related-protein), another primary receptor of ConA, is a super-glycosylated type-I transmembrane receptor with unknown possibly ligands. It has been found that adherent cells rely on the tyrosine phosphorylation of PZR for their detachment and migration. The intracellular section of PZR bears two immunoreceptor tyrosine-based inhibition motifs (ITIMs) that are binding sites for protein tyrosine phosphatases like SHP-1, SHP-2, and SHIP, which promote the inhibitory function of ITIMs. Upon activation of PZR receptors, they oligomerize and evoke Src family tyrosine kinases phosphorylating tyrosine amino acids of 241 and 263 in ITIMs, which recruit SHP-2. Whether the glycosylation profile of PZR is different in cancerous cells is not known yet, making it a precious target for modulation in the future [30, 31].

Beyond the two receptors mentioned above, it has been demonstrated that the biotinylated-ConA beads can pull down various RTKs (Receptor Tyrosine Kinases) involving INSR, IGFLR1, EGFR, and MET. It can also activate non-receptor RTKs such as PtdIns 3-kinase, GAP-associated P62, and some Src family kinases, which all are over-expressed in various tumor cells [32, 33].

Additionally, agglutination activity of ConA primarily originates from its binding to the glycoprotein II b – III, a complex at the surface of platelets, which induces their clustering and then stimulates their tyrosine-phosphorylation that is different and stronger than that created naturally by thrombin [34].

ConA can also act as an agonist of TLR-2 and TLR-6 (Toll-like receptor 2/6) trophic receptors and activate them in mesenchymal stem cells (MSCs), which leads to the up-regulation of Src and JAK/STAT3 signaling. It increases the transcription of colony-stimulating factors 1/2/3 (CSF1/2/3) [35–38].

## The molecular mechanisms of autophagy and apoptosis in cancer cells by ConA

In tumor cells, the expression level of RTKs is elevated, which also have a high proportion of β1,6-branched N-glycans. Unlike RTKs, growth-arrest receptors such as TGFβR1, TNFR1, and DR4, which have fewer N-glycan moieties, are down-regulated [39]. Therefore, in cancerous cells, the downstream signaling pathways of RTKs such as Ras-ERK, PI3K, and mTOR are significant levers controlling cellular responses such as proliferation, differentiation, motility, and survival or death to extracellular cues. As expected, most of these cells represent some degree of misregulation in these pathways [40].

ConA treatment predominantly targets PI3K/Akt signaling in tumor cells, where it remarkably reduces the level of phosphorylated Akt without altering the total Akt in several avenues [41]. In one way, oligomeric ConA binds to RTKs and triggers their granulation in the lipid rafts, which obsolete the conformational changes required for their activation without altering their ligand-binding capacity; Also, RTKs are no longer able to phosphorylate and initiate their related signaling cascades (e.g., PI3K/Akt, MAPK/ERK, and mTOR). In another way, P73 inhibits Akt phosphorylation following activation by ConA through unclear mediators. In Hella cells, ConA up-regulates MEK/ERK signaling and represses PI3K/Akt pathway. Furthermore, inhibition of Ras in PI3K/Akt signaling, known as the regulatory step between these two pathways, contributes to the higher activation of MEK/ERK signaling due to abrogating its inhibitory effect on this pathway [39–42].

Many human malignancies have been identified as lacking P53 or functionally inactivated through mutations. Several studies evidenced that ConA treatment selectively induces apoptosis in the cells with unfunctional P53, whereas those having normal P53, cancerous or not, are immune to the lethal features of ConA. In normal cells, indeed, without altering the P53 protein level, ConA induces its modification (like changing the phosphorylation status), which leads to P21/P27 induction and a transient cell-cycle arrest resulting in cellular repair [43]. Nonetheless, in the unfunctional-P53 cancerous cells, P73 plays a prominent role in controlling the cell fate by modulating various pro-and anti-apoptotic factors such as P21, Bax, Foxo1a, Bim, and Akt. The P21 induction might be P53-dependent or -independent and may bring about cell-cycle arrest both for pro-survival or pro-apoptotic purposes based on the type of stimuli [44, 45]. Following the up-regulation of P21 via ConA treatment, the inhibitory effect of CDK2 on Foxo1a, which is holding it in the cytoplasm, is blocked, leading to the nuclear localization of Foxo1a and subsequent activation of pro-apoptotic genes such as Fas, CD95, and Bid. The notion that P53 is the primary regulator of cell response to ConA treatment is consistent with the observation that the constitutive expression of Akt in P53-null cells could not prevent ConA-induced apoptosis. However, the P53 expression in these cells creates a full-protection, indicating that transrepression of Foxo1a by P53 is more significant than its post-translational modification by Akt [43, 45].

Many studies documented an apoptosis stimulation in different cell types treated with ConA. For instance, in human melanoma A375 cells, it has been shown that ConA treatment induces mitochondrial caspase-dependent apoptosis, where the up-regulation of caspase-3 and -9 occurs after cytochrome c is released into the cytoplasm. A significant decrease in caspase-activated DNAse (ICAD) inhibitor was also detected [46]. Moreover, in several human leukemia cell lines, it has been displayed that ConA could trigger apoptosis with an intrinsic pathway at low concentrations (5 µg/ml). In comparison, it can drastically elevate ROS production only at high doses (50 µg/ml). It should be noted that the ROS levels are not the initial apoptosis-inducing factor [47]. Hence, increasing the mitochondrial permeability and subsequent release of death factors were unrelated to the redox equilibrium or caspase-3 activation as the traditional mediators of apoptosis [40]. Instead, it is demonstrated that BNIP3, a BH-3 containing protein of the Bcl-2 family, mediates this event. It has a dual effect on the cell fate so that its cytoplasmic localization can suppress mTOR signaling, disturb pro-survival factors related to mitochondrial function (apoptosis), and provoke LC3- II formation (autophagy). On the other hand, its nuclear localization can enhance the expression level of pro-survival genes to increase cell survival; the earlier induced as a result of stress conditions and the latter by hypoxia [48–50].

Likewise, several lines of evidence have suggested that ConA can trigger autophagy response primarily through enhancing JAK2/STAT3 signaling. The current evidence indicates that the NANOS1 protein is the first effector activated by ConA, a vital protein controlling cell migration via regulating the MT1-MMP mRNA and protein expression. Remarkably, the NANOS1 expression rate is also negatively regulated by the E-cadherin adhesion molecule level [51]. Of note, how ConA recruits the NANOS1 proteins is not yet known. However, NANOS1 could significantly elevate the BNIP3 expression and cause its cytoplasmic localization once recruited. Upon the translocation, BNIP3 increases mitochondrial pore formation and interacts with LC3-I, which the latter lipidated to LC3- II and triggers autophagy by binding to autophagosomes [50]. To date, requiring any interaction between MT1-MMP and JAK2 is unknown. Notably, autophagy and apoptosis are tied in many aspects; apoptosis might be modulated or elevated via autophagy. When it is hampered (e.g., in tumor cells), autophagy could commence cell death. Interestingly, it has been revealed that by inhibiting autophagy with its inhibitor 3MA in Hella cells, ConA could not induce autophagy and was not able to perform apoptosis, suggesting the contributory role of autophagy in cancerous cells [42].

Studies using GFP fluorescent tags revealed that ConA could directly bring about hepatoma cell death in an autophagic manner. The bound-bead lectin could not induce LC3- II formation. The results showed that the lectin also needs to be internalized and then assembled on mitochondria to activate BNIP3, which is followed by disturbing the mitochondrial permeability, in turn, releasing some lethal factors to the cytoplasm/nucleus involving cytochrome c, AIF, and Endo G. Besides, BNIP3 activation also contributes to LC3- II formation which triggers autophagy. Confirming this, either beclin-1 or the ATG5 siRNA but not LC3 siRNA failed to prevent ConA-induced cell death, proposing a BNIP3-mediated mitochondria autophagy [48].

In addition to mitochondria and lysosomes as the principal initiators of apoptosis and autophagy, ER is also considered one of the leading originators of cell death. It acts as a sensor of cellular stress via modulating Ca^2+^ influx by G6PT (glucose-6-phosphate transporter), which is a microsomal resident protein that acts as a pro-survival factor through an ATP-consuming process that contributes to Ca^2+^ sequestering from the cytosol to ER, so it can sense ATP or G6P depletion and begin cellular apoptosis [41]. The G6PT expression level is mainly high in many cancers, possibly because of its pleiotropic intracellular functions, which implied from this observation that its overexpression prevents the ConA-stimulated MMP-2 activation but does not repress the ConA-induced cell death. Also, it has been reported that the ConA treatment or MT1-MMP overexpression can lead to G6PT down-regulation [41]. The increased calcium influx mediated by this repression impedes the localization of MT1-MMP and subsequent activation of MMP-2, preventing tumor cell metastasis.

**Fig. 2:**
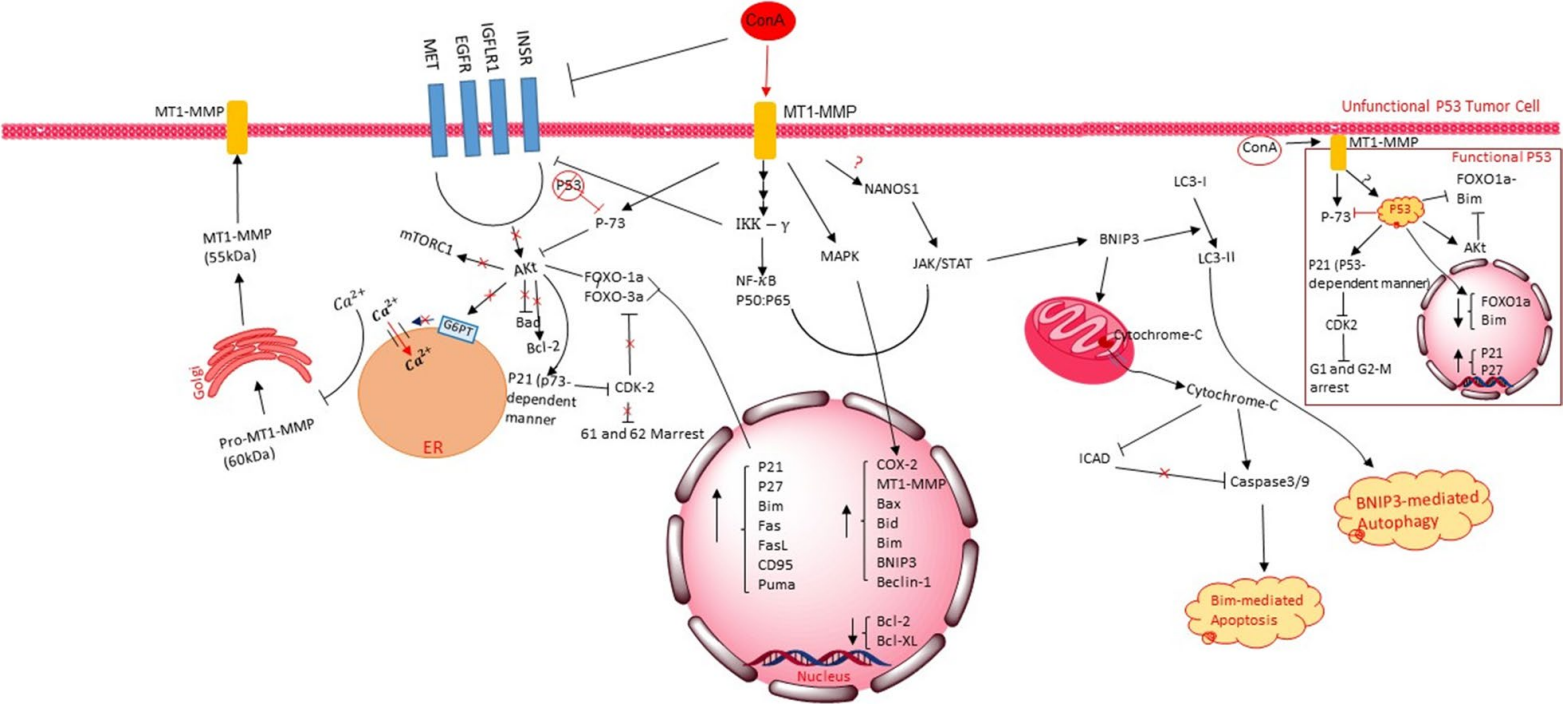
The illustration of the possible signaling circuits involved in MT1-MMP-mediated autophagy and apoptosis following ConA treatment. In the unfunctional-p53 tumor cells, ConA can induce P73, which leads to the translocation of FOXO transcription factors to the nucleus so that they can induce an apoptotic gene profile. Also, ConA can induce JAK/STAT-3, MAPK, and NF-κB signaling pathways, which all would carry on the autophagy and apoptosis processes by modulating some essential factors like inducing BNIP3 to exit from the nucleus and initiate both programmed cell death I and II. Moreover, via inhibiting the Akt signaling pathway, ConA can increase the cytoplasmic level of Ca^2+^, which results in the accumulation of unmatured MT1-MMP in the cytoplasm of cancer cells. In P53-containing cells, nevertheless, ConA activates p53 in a post-translational way to create a transient cell-cycle arrest, which will induce cellular DNA repair

Hence, ConA modulates several downstream signaling pathways with a broad spectrum of consequences, from hindering tumor cell proliferation and triggering programmed cell death I/II to facilitating dead cell obliteration by the immune system (Fig. 2 and Tables 1 and 2). It could trigger apoptosis mainly by down-regulating Akt phosphorylation, but not in functional P53 tumor cells. However, ConA causes cancer cell death predominantly by inducing autophagy. It partially induces JAK/STAT signaling through some unknown mechanisms, resulting in BNIP3 expression and translocation. Then BNIP3 can carry on both the autophagy and apoptosis processes. Scant plant lectins induce autophagy and apoptosis in targeted cells, making ConA a vigorous anti-cancer agent.

**Table 1:** Specific mechanisms and target effectors involved in the ConA-mediated- apoptotic or autophagic cell death, hepatitis, fibroblast activation, and angiogenesis (in vitro studies)

Cancer cell type	ConA concentration (µg/ml)	Target(s)	The specific mechanism(s)	References
KK-47 bladder	0.2–20	DNA fragmentation, cytoskeleton reorganization	Inhibiting RNA synthesis	[15]
U87 glioblastoma	30	LRP-1	LRP-1 down-regulation and mistrafficking, disrupt cytoskeleton integrity	[17]
Human melanoma A375	25	Caspase 9 and 3, cyto.c, ICAD, PARP	Caspase-dependent apoptosis	[18, 46]
Glioma cell lines (UWR2, UWR3, U2S1MG, and SNB-19)	100	Gelatinase A, MT1-MMP	Inducing malignant progression by MT-MMP/MMP2 up-regulation	[21]
3Y1	5–20	c-Ras, MT1-MMP, MMP-2/9	c-Ras-dependent activation of the MMPs	[23]
U87 human glioma cells, COS-7	10	MT1-MMP, pro-MMP2	pro-MMP2 activation through the proteolytic activity of MT1-MMP	[24]
MDA-MB-231	20	proMT1-MMP	Inhibiting MT1-MMP maturation by decreasing calcium levels	[25]
MDA-MB-231	20	MT1-MMP, proMMP2	Increase MMP2 levels by inducing tyrosine phosphorylation	[27]
HEK293T and HSC-4	30	MT1-MMP, HAI-1, Matripase	Increase matriptase protease activity through the cleavage of HAI-1	[28]
Mesenchymal stromal cells(MSC)	30	MT1-MMP, STAT3	Increase COX2 expression via MT1-MMP/JAK/STAT3 signaling	[29]
Bone marrow-derived dendritic cells (BMDC)	10	ALT, AST, IL-12, IFN-γ, P62	Increase the maturation of BMDCs by aberrant regulation of autophagy, thereby augmenting cytokine secretion	[33]
Mesenchymal stromal cells(MSC)	30	MT1-MMP, STAT3, Src	Up-regulate CSF1-2-3 secretion through TLR-2/6 activation	[36]
HeLa, Caco-2, and A549	2–50	BAD, Bcl-2, CASP-3/9, AKT	Inhibiting the receptor of tyrosine kinases by aggregating them in lipid rafts and inducing apoptosis	[39]
U87 glioblastoma	10	MT1-MMP, MMP2, G6PT, Akt	Inhibiting G6PT and Akt by MT1-MMP cytoplasmic domain, inducing apoptosis	[41]
HeLa	54	Beclin-1, LC3-I/II, Akt, MEK	Inducing autophagy by activating PI3K/Akt/mTOR and MEK/ERK pathways	[42]
SKOV3, MDAH041, SKP53, and TR9-7	15–20	FOXO1a, Bim, p53, p21, p27	Inducing apoptosis by activating FOXO1a-Bim signaling	[43]
MCF-7 and MCF-10A	1–100	Caspase 9 and 3, Cyto.c, Bax, Bid, Bcl-2, Bcl-X_L_, NF-κB, ERK, JNK, p53, p21, CDK-1/2	Inducing caspase-dependent apoptosis	[44]
MDAH041, TR9-7, and SKOV3	15	Bax, Bcl2, p73, p21, Foxo1a, Bim, Akt	Inducing p73-mediated apoptosis	[45]
Human leukemia MOLT-4 and HL-60	1–200	DNA fragmentation, cytoskeleton reorganization	Inducing apoptosis	[47]
ML-1, CT-26, Huh-7, and HepG2	7.7–20	Beclin-1, ATG5, LC3-I/II, BNIP3, Akt, COX-4	Inducing BNIP3-mediated autophagy	[48]
U87 glioblastoma	30	MT1-MMP, MMP2, NANOS1, BNIP3, STAT3, ATG-3/12/16L1/16L2, COX2, Akt	Inducing autophagy through the intracellular domain of MT1-MMP	[49–54]
Human umbilical vein endothelial and Ea.hy926 endothelial cells	0.3–3	Akt, ERK, p21, p27, p38, Cyclin D1, Cyclin E	Promoting angiogenesis through Akt/ERK/Cyclin D1 axis	[55]
BALBlc 3T3 fibroblasts, human gingival fibroblasts (HGF)	50	DNA fragmentation, cytoskeleton reorganization	Cell cycle arrest, inhibiting DNA and RNA synthesis	[56]
Human fibroblast and COS7	20–50	TIMP-2, SHP-2, ERK, p38, MMP2, Ras, SOS-1, Grb-2	SHP-2-mediated upregulation of TIMP-2, thereby fibroblast proliferation	[57–59]
CD4+ T cells	10	IFN-γ, TNF-α, IL-4/6/10/12, STAT-1/3, p65	CD24 aggravates ConA-induced liver injury	[72]
Liver mononuclear cells	10	IFN-γ, TNF-α, IL-4, FasL	Va14 NKT Cells develop ConA-induced hepatitis through IL-4 production	[73]
ML-1, HuH-7, and Hep G2	20	LC3-I/II, Casp3, STAT3, MIF, BNIP3	Inducing STAT3-MIF-BNIP3-mediated autophagy in the hepatoma cells	[90, 91]

**Table 2:** Specific mechanisms and target effectors involved in the ConA-mediated- apoptotic or autophagic cell death, hepatitis, fibroblast activation, and angiogenesis (in vivo studies)

Experimental model(s)	ConA concentration	Target(s)	The specific mechanism(s)	References
C57BL/6 mice	20 mg/kg	ALT, AST, IL-12, IFN-γ, P62	Increase the maturation of BMDCs by aberrant regulation of autophagy, thereby augmenting cytokine secretion	[33]
Balb/C mice. mice PBMC	10 mg/kg, 10 µg/ml	TNF-α, IFN-γ, IL-6, NF-κB	Increase inflammatory cytokines by TLR-2 stimulation	[37]
Breast carcinoma MCF-7 bearing nude mice	40 mg/kg	Caspase 9 and 3, Cyto.c, Bax, Bid, Bcl-2, Bcl-X_L_, NF-κB, ERK, JNK, p53, p21, CDK-1/2	Inducing caspase-dependent apoptosis	[44]
Severe combined immune deficiency (SCID) and BALB/c mice	7.7–20 mg/kg	Beclin-1, ATG5, LC3-I/II, BNIP3, Akt, COX-4	Inducing BNIP3-mediated autophagy	[48]
Hind-limb ischaemic mice	10 mg/kg	Akt, ERK, p21, p27, p38, Cyclin D1, Cyclin E	Promoting angiogenesis through Akt/ERK/Cyclin D1 axis	[55]
C57BL/6 mice	10–15 mg/kg	IFN-γ, TNF-α, IL-2/6/10	Elevating tolerogenic state mediated by IL-10, Treg, and Kupffer cells	[66]
C57BL/6J, BALB/cJ mice	10–22 µg/g	JAKs, STATs, SOCSs, p21, p53, Casp-3, Bcl-2, Bcl-X_L_	Inducing hepatitis via activating STAT3 signaling	[67]
BALB/c mice	0.05 mg/200 µL PBS	ALT, AST, IFN-γ	Inducing hepatitis by activating NK, NKT, CD4^+^ and CD8^+^ T cells	[68]
BALB/c mice	0.5 µg/0.5 ml PBS	TNF-α, IL-4/10	Alleviation of liver injury by stimulating anti-inflammatory cytokines	[69]
BALB/c mice	10–20 mg/kg	IFN-γ, TNF-α, IL-4/6/10/12, STAT-1/3, p65	CD24 aggravates ConA-induced liver injury	[72]
C57BL/6 (B6) mice	25 and 37.5 mg/kg	IFN-γ, TNF-α, IL-4, FasL	Va14 NKT Cells develop ConA-induced hepatitis through IL-4 production	[73]
C57BL/6 mice	3 and 15 µg/g	IFN-γ, TNF-α, IL-2/4/6/10/12	Differential effect of low and high dose ConA on cytokine profile	[74]
BALB/c and C57BL/6J mice	12 and 15 mg/kg	STAT4, IL-12A/12B, FasL	Inducing STAT4 activation in inflammatory cells contributes to liver injury alleviation	[75]
C57/BL6 mice	10 and 12 µg/g	ALT, AST, IL-22, IL-22R, STAT-1/3, ERK-1/2	Stimulating IL-22 contributes to hepatocyte survival by STAT3 activation	[77]
BALB/c mice	10 and 20 mg/kg	IFN-γ, TNF-α, IL-4/10	ConA treatment prevents hepatitis by inducing Tregs	[78]
C57BL/6 mice	20 mg/kg	ALT, AST, IFN-γ, TNF-α, IL-6/17/1β, TGF-β	Inducing Kupffer cells through Th1-type response mediates liver injury	[85]
BALB/c mice	10 mg/kg	LC3-I/II, Casp3, STAT3, MIF, BNIP3	Inducing STAT3-MIF-BNIP3-mediated autophagy in the hepatoma cells	[90, 91]

## Molecular modulation of angiogenesis by ConA

By attracting MSCs and transforming them into tumor-associated fibroblasts, metastatic cells can induce angiogenesis, where MSCs secrete CSFs (colony-stimulating factors) [36]. Several studies have revealed that ConA could inhibit angiogenesis through the up-regulation of the COX-2 level (Tables 1 and 2). Reportedly, MT1-MMP is also the upstream regulator of the COX-2 inflammatory protein expression via KF-κB/IKK proteins as the primary mediators [52]. Two kinases of IKK-α and IKK-β plus regulatory subunit IKK-γ constitute the IKK complex, which is essential for all the inducible NF-κB signaling pathways [53]. In U87 glioma cells, different concentrations of ConA revealed a positive linear correlation between MT1-MMP and COX-2 expression levels. Also, an inverse correlation between the degree of Akt phosphorylation and COX-2 expression was observed, leading to angiogenesis inhibition. Studies also displayed that the NF-κB p50 and IKK-γ are mandatory for the ConA-mediated COX induction through the intracellular domain of MT1-MMP. In these cells, COX-2 overexpression induced by ConA also correlates with the GRP78 protein expression, an indicator of ER stress [53, 54].

Nevertheless, it should be noted that in an opposite report, angiogenesis stimulation by ConA has also been shown. It revealed that the ConA administration remarkably induces human endothelial cell proliferation and angiogenesis via an Akt/ERK/cyclin D1 axis and augments the secretion of pro-angiogenic factors such as VEGFa PDGFaa, and bFGF. The pro-angiogenic effect of ConA has also been proved in the hind-limb ischemia mice, where ConA promoted fixing the ischemia hind-limb. However, the exact receptors responsible for this mitogenic/angiogenic effect remain to be further elucidated. These findings open a new insight on the ConA potential as an anti-neoplastic agent that might arise different responses in different cells, whether or not by modulating the same signaling pathways. Therefore, the lectin can even be used as an anti-atherosclerosis agent to repair myocardium in acute MI (myocardial infarction) [55].

## Molecular mechanisms of MMP-2 production in fibroblasts by ConA

**Fig. 3:**
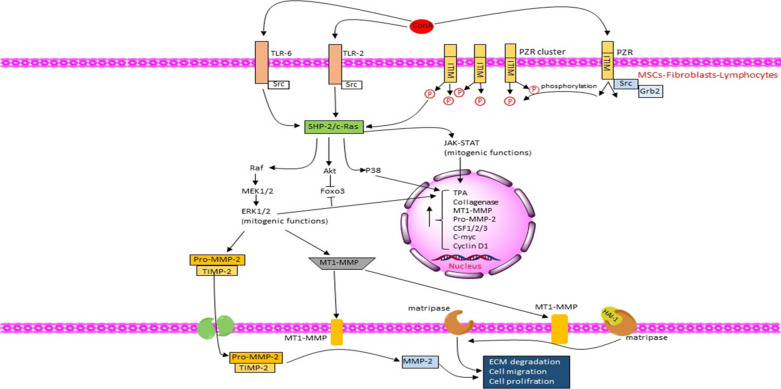
Cartoons represent the molecular mechanism of the proliferative effects induced by ConA in fibroblasts, lymphocytes, and MSCs. The lectin acts as an agonist for TLR-2, TLR-6, and PZR receptors, which all induce the SHP-2/c-Ras axis as their main downstream effector following ConA treatment to activate the mitogenic functions of STAT3 and ERK1/2 signaling molecules

In tumor cells, the synthesis and secretion of MMP-2 and TIMP-2 are in charge of the neighboring stromal fibroblasts [56]. The secretory proteins of TIMP-2 have a dual influence on the MMP-2 activity; in low concentrations, they act as mandatory adaptors in the proteolytic activation of pro-MMP-2, while in high amounts, they act such an inhibitor for the MMP-2 activation [57]. ConA stimulates MMP-2 expression in human fibroblasts and reciprocally decreases the TIMP expression. Further, it can directly augment collagenase synthesis to degrade ECM and elevate the synthesis of plasminogen activators in macrophages, stimulating collagenase synthesis through a positive loop [21, 58]. Experiments in dominant-negative ras (S17N ras) 3Y1 cells demonstrated that c-Ras is pivotal for MMP-2 activation by MT1-MMP, where MT1-MMP expression was abolished in cells with the negative ras. Therefore, ConA treatment strengthens pro-MMP-2 in fibroblasts by activating a pre-existing pool of MT1-MMP, then boosting its transcription [24]. Even though the key factors determining the MT1-MMP pro-apoptotic or pro-survival signaling remain to be clarified, SHP2 (SH2 containing protein tyrosine phosphatase-2) induction might be an indispensable element. SHP2 is a tyrosine phosphatase primarily expressed in the cytoplasm and is known to up-regulate ERK and Akt mitogenic cascades. It forms a complex with the other constituents of the cascade (e.g., Grb-2 and SOS-1) and serves as a connector between growth factor receptors (tyrosine phosphorylation) and Ras-MAPK signaling [59, 60]. Intriguingly, an interaction between SHP2 and PZR receptors also deciphered, and ConA exposure, extensively and in a time- and dose-dependent manner, induces PZR clustering then SHP2 recruitment [30]. In addition, studies have shown that the SHP2/Ras-dependent activation of ERK1/2 and P38 is a prerequisite for the functional MMP-2 activation and secretion following ConA administration. Consistent with the finding that SHP-2 is the primary factor exerting mitogenic influences, it has also revealed that the SHP2-mutated cells were incapable of producing and secreting MMP-2 mainly due to the impaired ERK1/2 and the P38 activation in the mutant cells. Hence, the reintroduction of the wt-SHP2 resulted in the reactivation of MMP-2 secretion. Interestingly, constitutive MEK1 expression in SHP2-mutant cells failed to rescue MMP-2 activation, suggesting the essential role of both ERK and P38 in the proteolytic activation of MMP-2. Nonetheless, ConA exposure in these cells stimulated MMP-2 production via constitutive ERK signaling and the P38 up-regulation (Fig. 3 and Tables 1 and 2) [59].

## ConA modulates immune cells function and signaling, resulting in liver injury

In addition to death induction in various cancer cell types, ConA has also been shown to act as a cell proliferation activator in some other non-cancerous cell types. This feature of mitogenicity and, simultaneously, inducing tumor cell apoptosis in a dose-dependent manner in various cell lines makes such lectins precious agents in cancer therapy. The combined evidence to date suggests that the mitogenic properties of ConA are primarily advent at the doses of 1–10 µg/ml, and induction of autophagy and apoptosis at > 20 µg/ml, while > 50 µg/ml are cytotoxic doses [61]. The liver is an ideal site for the ConA assembly because of its anatomic location as the first place where blood-born alien substances encounter. ConA injection revealed differential activities on hepatoma cells. It can inhibit tumor nodule formation by inducing autophagy on the one hand and stimulating lymphocytes to kill hepatocytes on the other hand. Unfortunately, lymphocytes are more prone to ConA activation than hepatoma cells to ConA inhibition [48].

Of note, since activation of lymphocytes needs receptor clustering, multivalent lectins like ConA are in great attention for their potential mitogenic properties. By assessments of different oligomers of ConA, which have been accomplished by the merci of various chemical modifications and protease administrations, it has been shown that just the multivalent types of ConA can trigger their associated signaling cascades. For instance, succinyl ConA, the un-oligomerized variant of ConA, has been shown unable to induce MT1-MMP signaling, indicating the importance of receptor clustering in transducing biological signals [23, 62]. Importantly, receptor-tyrosine phosphorylation is the central hub in important cellular events as if proliferation, differentiation, and transformation mediated by various extracellular stimuli. As shown by in vitro evaluations, protein tyrosine phosphorylation in both proliferative lymphocytes, e.g., B- and T-cells, and non-proliferative ones, e.g., neutrophils and platelets, have been induced upon the ConA administration through the JAK/STAT3 signaling pathway [63–65].

### ConA administration activates both pro- and anti-inflammatory cytokines

Activating a broad range of lymphokines has been reported as the result of mitogenic effects of ConA [66]. It has been displayed that the liver injury caused by ConA injection was mediated primarily by NK- and NKT-cell activation, CD69 expression, and stimulation of inflammatory cytokines from CD4+ and CD8+ T cells [67, 68]. This cytokine storm has been evident to be the fundamental reason for liver injury as the most severe side-effect of the pre-clinical usage of the lectin. Cytokines such as TNF-α, IFN-γ, IL-1, IL-6, and IL-12 are in the inflammatory group, while IL-4, IL-10, and IL-22 are anti-inflammatory cytokines [69–71]. TNF-α acts as an apoptosis facilitator, whereas IFN-γ serves as a macrophage or NK cell proliferator; both increase the MHC- II expression, contributing to liver injury. In addition to causing hepatocellular damage, stimulated T-cells also have protective roles via producing anti-inflammatory and growth-inducing cytokines as though IL-4, IL-10, and IL-22, which act by activating MAPK and JAK/STAT3 signaling pathways. Moreover, it has been revealed that non-hepatotoxic low doses of ConA injection in the murine models can exert its anti-tumor effects, mainly by activating NK cells [69].

Several studies have reported that the CD4^+^T cells, CD8^+^T cells, and NKT cells are the main drivers of ConA-induced liver injury; however, other immune cells involving neutrophils, kupffer, and B cells are also detected in the liver upon the lectin administration [72]. CD4+ T cells are likely the most significant contributor to the three main hepatic injury drivers. CD24 is primarily expressed in normal stable situations on the non-T lymphocytes controlling their homeostasis, but, ConA has been shown to induce its expression on the hepatic T cells. In this regard, ConA injection in mice (10–20 mg/kg) has been reported to elevate the production of IFN-γ and TNF-α by CD4+ T cells leading to acute liver injury. Moreover, CD24 deficient mice showed a lower liver injury, which resulted from a decrease in the secretion of inflammatory cytokines by CD4+ T cells with no observed difference in the activation or number of other mononuclear lymphocytes (like NKT and CD8+ cells). Experiments also demonstrated a reduction in the STAT1 phosphorylation level in CD24 deficient mice. Whereas, upon the CD24 activation in the normal T cells, IFN-γ caused the translocation of STAT1 to the nucleus, where it up-regulates genes that promote liver injury and inflammation. The secreted IFN-γ is also the central priming factor of macrophages that enhances TNF-α production leading to hepatocyte cell death [72]. In the case of NKT cells, they predominantly secrete IL-4 and IL-5, which in turn recruits eosinophils causing liver injury [73].

There are also studies indicating the significant potential of ConA in inducing anti-inflammatory cytokines. In an in vivo study that used BALB/c mice as the murine model of liver injury, the concentrations of TNF-α inflammatory cytokine and IL-4 and IL-10 anti-inflammatory cytokines, following ConA injection, concomitantly increased. The inflammatory cytokine had induced liver injury, hinted at by an escalation in AST and ALT transaminases after 8–24 h exposure [69]. Another in vivo study on a murine model recently has shown that even though the low and high doses of ConA both stimulate CD3+ and NK cells in the liver, each one cause a distinctive cytokine expression profile; where the non-hepatotoxic amounts of the lectin (3–5 µg/g) induced IL-6 expression with no impact on IL-4, IL-12, TNF-α, and IFN-γ, while the hepatotoxic doses (15–20 µg/g) showed an augmentation of these inflammatory cytokines. Furthermore, the intrahepatic Fas-FasL system induced by high doses led to hepatocyte injury, while pre-treatment with low doses prevented the lethal effects of hepatotoxic doses [74]. The bulk of studies suggested FasL-mediated apoptosis by NKT cells as the leading cause of ConA-mediated liver injury. Despite the necrosis created by high doses of ConA, its low doses trigger IL-2 production by T cells, NKT cells, macrophages, and kupffer cells (KCs), which exerts its anti-inflammatory effect by STAT4 activation. This activation has two distinctive impacts on T-cell hepatitis. It promotes Th1 and Th2 (T helper) cytokines as its minor role that leads to IFN-γ production, while its major comes from inhibiting FasL in NKT cells, which attenuates the acute T-cell hepatitis [75, 76]. Corroborating the induction of protective interleukins, some recent evidence revealed that following the ConA injection (10 µg/g), IL-22 considerably up-regulated at both the mRNA and protein levels, mainly in the CD3+ cells. The expression level of IL-22R was enhanced in hepatocytes as well. This study also revealed that the pre-treatment with IL-22 antibodies exacerbated liver necrosis, while pre-exposure with rIL-22 prevented liver injury and ALT/AST escalations. IL-22 activates the mitogenic functions of STAT-3, which subsequently stimulates anti-apoptotic and growth-relating genes. Thus, IL-22 could be exploited as an adjuvant drug to relieve the ConA-induced liver injury [77].

Other T cell subsets with suppressive functions stimulated via ConA treatment are regulatory T cells (Tregs). They are non-proliferative cells that mainly exert their immune tolerance by producing IL-10. In vivo ConA pre-treatment has stimulated Tr1 cells with elevated CD69 and CD44 expression. Thus, clinical targeting of Tr1 cells might be considered an efficacious approach to mitigate the hepatocyte injury. Liver sinusoidal endothelial cells (LSEC) and KCs are additional cell types responsible for ConA-mediated IL-10 production [78–82]. Along with anti-inflammatory activities, KCs can also produce pro-inflammatory cytokines such as IFN-γ, TNF-α, and IL-6. Furthermore, after ConA binding to KC receptors, Th1, and Th2 cells can recognize MHC class II on KCs, followed by more inflammatory cytokine production [83, 84]. Collectively, ConA first activates KCs in the liver to secrete pro-inflammatory cytokines, leading to a Th1 type response within the liver; the CD4+ and NKT cells enhance the generation of inflammatory cytokines. The produced cytokine storm finally contributes to hepatoma injury. However, pre-treatment with low concentrations of ConA predominantly induces the anti-inflammatory profile of cytokines, protecting the liver from injury [85].

### ConA activates NANOS1/MIF/BNIP3 cascade through STAT3 induction in hepatocytes

In the T cell activation process, Ca^2+^ channels play a central role. TRPA1 (transient receptor potential subfamily A member 1) is a cationic channel at T cells' cell surface permeable to calcium ions. Upon activation, it drives Ca2 + influx, which regulates several transcription factors such as NFAT, NF-κB, and JNK leading to the secretion of effector cytokines, predominantly IFN-γ, TNF-α, and IL-2. It has been shown that ConA treatment stimulates TRPA1 expression in T cells. Also, implementing the TRPA1 inhibitor abrogated ConA-induced T cell activation by reducing the expression of CD25 and CD69 activation markers and the signature effector cytokines. These observations propose a possible role for TRPA1 in initiating the molecular mechanisms that eventuate T cell activation [86].

Further studies have shown that ConA exposure for 48 h (10 µg/ml) can induce pro-inflammatory cytokines such as TNF-α, IL-1β, and IFN-γ in lymphocytes, which do activate iNOS leading to multiplying lymphocytes [87]. Also, ConA injection induces STAT1/3 in lymphocytes resulting in their proliferation. It has been unveiled that STAT-1 activates IFN-γ, but STAT-3 shows a protective role in the liver injury mediated by T-cells. STAT-1 and -3 have a mutual antagonism relationship mediated by SOCS3 proteins, induced by both STATs, but in return, SOCS proteins inhibit them by down-regulating JAKs. In this respect, STAT-3 seems more potent than STAT-1 in causing SOCS3 (in the ConA-injected mice), potentially contributing to liver regeneration by regarding the protective role of STAT-3 [67]. Remarkably, STAT3 has a dual impact on cell proliferation. It can elevate the expression of mitogenic genes (e.g., c-myc and cyclin D1), and conversely, it can activate genes mediating cell cycle arrest (e.g., P21cip/WAF) [77]. Moreover, the mitogenic effect of ConA-induced STAT3 in lymphocytes has also been associated with activating iNOS (inducible Nitric Oxide Synthase), which induces cell proliferation via increasing NO signaling [87].

In hepatocytes, NANOS1 acts as a mediator in STAT-3 induction by ConA. In the conditions where either NANOS1 or STAT-3 was silenced, the gene and protein expression of MT1-MMP was abrogated. By silencing NANOS1 gene expression merely, STAT-3 phosphorylation after ConA treatment was abolished, suggesting the essential role of NANOS1 as an upstream regulator in triggering ConA signaling. However, the detailed mechanism of how ConA stimulates NANOS1 transcription remains unclear [51].

At first, MIF (Macrophage migration inhibitory factor) was explored as a pleiotropic cytokine that inhibits macrophage random migration. It is primarily released from T-cells and at low levels in others such as endothelial, epithelial, hepatocytes, and cancerous cells. After secretion in stress conditions, MIF can act in both para- and autocrine manners via binding to its receptor CD74, which triggers downstream signaling resulting in BNIP3-dependent autophagy [88–90]. As expected, KO mice, which have no expression of MIF mRNA, treated with ConA are resistant to ConA-induced liver injury, likely due to the abrogation of TNF-α/IFN-γ stimulation by MIF [91]. ConA also induces STAT-3 phosphorylation, and following COX-2 expression, without changing total STAT-3, occurs through JAK and MEK signaling. In MAPK signaling, similar to STAT3, ERK1/2 might cause hepatocyte proliferation or arrest, depending on the amount of activated protein [77]. Furthermore, the gene silencing of MT1-MMP abrogates STAT-3 and pro-MMP2 activation plus COX-2 expression. Interestingly, this silencing did not impact the STAT-3 phosphorylation induced by IL-6, proposing a link between ConA and MT1-MMP [29].

**Fig. 4:**
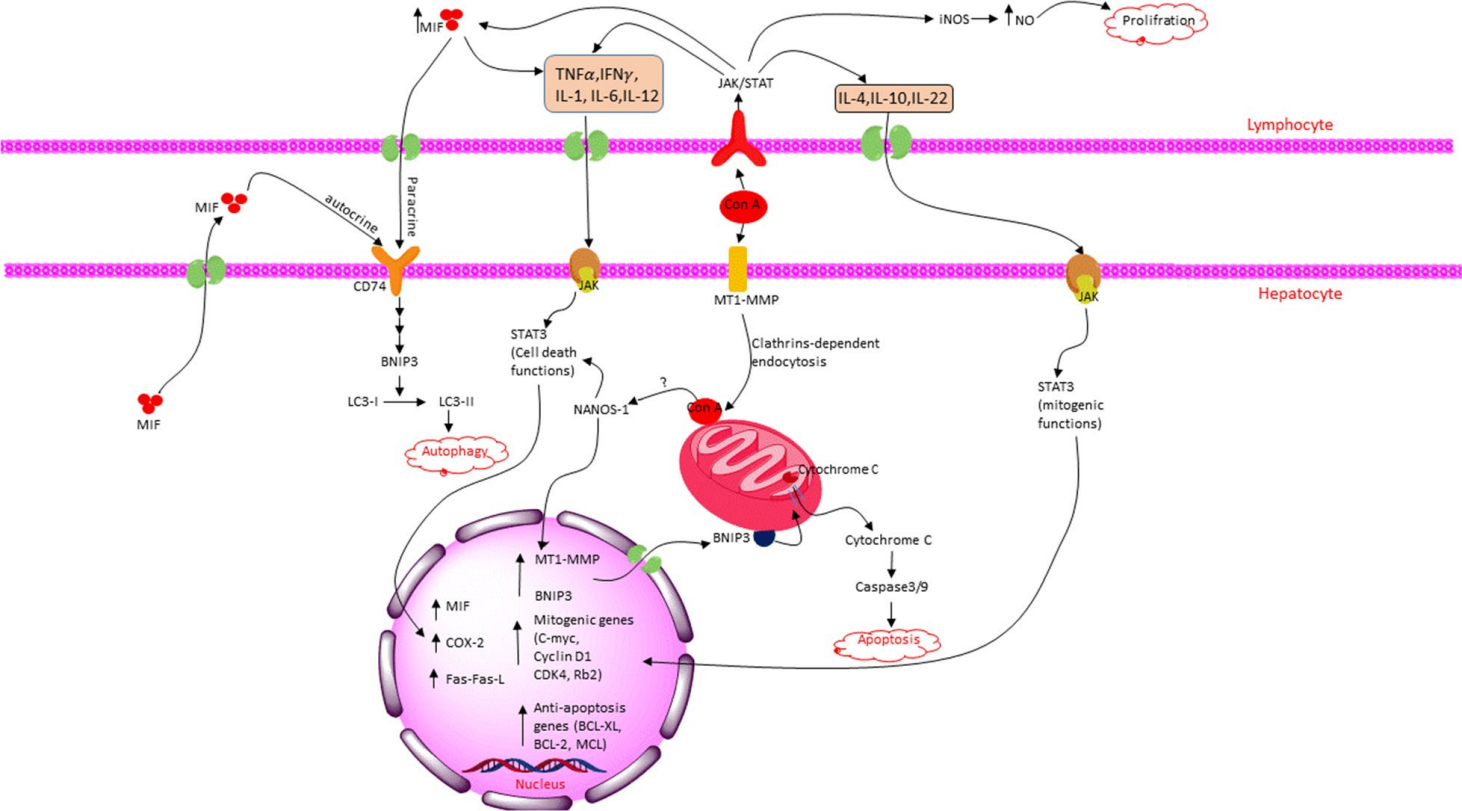
Schematic representation of the crosstalk between activated lymphocytes and hepatocytes, following the ConA administration, thereby liver injury. On the one hand, the inflammatory cytokines secreted from the ConA-induced lymphocytes would induce the cell-death functions of STAT3 in hepatocytes, which activates BNIP3, then autophagy and apoptosis induction. On the other hand, the anti-inflammatory cytokines would stimulate mitogenic functions of STAT3, leading to the up-regulation of mitogenic and anti-apoptosis genes

In hepatoma cell lines and in situ hepatoma murine models, the receptor-mediated endocytosis of ConA is necessary for STAT-3 translocation to the nucleus and transducing its signals. Several studies have clarified the downstream order of the modulated signals upon NANOS1 induction by ConA, and it has revealed that by inhibiting STAT-3 phosphorylation via Static, BNIP3, and LC3- II stimulation would decline. Nevertheless, inhibiting MIF via ISO- I cause BNIP3 down-regulation without any alteration of STAT-3 phosphorylation status, suggesting a STAT-3/MIF/BNIP3 arrangement to stimulate LC3- II conversion and triggering autophagy (Fig. 4 and Tables 1 and 2) [90]. Deciphering the whole and complex network of ConA downstream signaling circuits is likely to provide new mechanistic insights into how the various responses created by the lectin are regulated; As well as, it will enable us to develop new approaches to safely benefit from ConA as a drug in many fields of medicine.

## Conclusions and outlook

Because of the crosstalk between downstream effectors of RTKs, there are two approaches to prevent drug resistance; using several inhibitors for different RTKs and serving one agent for inhibiting multiple RTKs. Lack of specificity and toxicity are limiting factors of these strategies. As discussed in this article, preferential binding to cancerous cells and simultaneously deactivating various RTKs, are privileges of ConA lectin, making it a robust anti-cancer candidate. Likewise, ConA can induce autophagic and apoptotic cell death in tumor cells by binding to several receptors involving MT1-MMP and RTKs, modulating their downstream signaling pathways such as PI3K/Akt, JAK/STAT, MAPK, and NF-κB [39].

Nevertheless, there are some disadvantages of using ConA for clinical purposes in the future. MMP activation is a prerequisite process of metastasis in tumor cells. Its stimulation by ConA treatment is a dark side of its usage as an anti-tumor drug; logically, the matrix metalloproteinase blockers would be required for a more efficient anti-cancer therapy. Additionally, following the five or six ConA administrations, anti-ConA antibodies would be produced and elevated. Note that antibody arising is the common drawback of protein drugs that hinder their repetitive exploitation. Hence, it puts tremendous pressure on deciphering novel lectins, whose anti-cancer properties and cytotoxicity profiles are close to each other, enabling us to administer them sequentially [48, 92].

Moreover, the observations of liver injury in pre-clinical trials limited the direct usage of ConA as an anti-neoplastic drug. Shortly after ConA injection, a cytokine storm creates, mainly mediated by CD4+, CD8+, and NKT cells. However, pre-treatment with low doses of the lectin induces a different cytokine profile, mainly constituted of anti-inflammatory cytokines, endowing us with precious insights for medical prevention. Therefore, to harness this strong lectin for cancer therapy in the clinical phase, we should eradicate its significant downsides, including toxicity and glycoprotein nature. In this regard, site-directed mutagenesis and artificial peptides, as used for boosting other glycoprotein drugs or lectins, are exiting approaches to alleviate its cytotoxicity without a harmful impact on the functionality of the lectin and making it clinically lucrative [93, 94].

## Data Availability

No data was used for the preparation of this manuscript.

## References

[1] Clark D, Mao L (2012). Cancer biomarker discovery: lectin-based strategies targeting glycoproteins. Dis Markers.

[2] Badr HA, AlSadek DM, Darwish AA, ElSayed AI, Bekmanov BO, Khussainova EM, Zhang X, Cho WC, Djansugurova LB, Li C-Z (2014). Lectin approaches for glycoproteomics in FDA-approved cancer biomarkers. Expert Rev Proteomics.

[3] Yu H, Shu J, Li Z (2020). Lectin microarrays for glycoproteomics: an overview of their use and potential. Expert Rev Proteomics.

[4] Poiroux G, Barre A, Van Damme EJ, Benoist H, Rougé P (2017). Plant lectins targeting O-glycans at the cell surface as tools for cancer diagnosis, prognosis and therapy. Int J Mol Sci.

[5] Tsaneva M, Van Damme EJ (2020). 130 years of plant lectin research. Glycoconj J.

[6] Nonis SG, Haywood J, Schmidberger JW, Mackie ER, Soares da Costa TP, Bond CS, Mylne JS (2021). Structural and biochemical analyses of concanavalin A circular permutation by jack bean asparaginyl endopeptidase. Plant Cell.

[7] Jiang QL, Zhang S, Tian M, Zhang SY, Xie T, Chen DY, Chen YJ, He J, Liu J, Ouyang L (2015). Plant lectins, from ancient sugar-binding proteins to emerging anti-cancer drugs in apoptosis and autophagy. Cell Prolif.

[8] Cavada BS, Pinto-Junior VR, Osterne VJ, Nascimento KS (2019). ConA-like lectins: high similarity proteins as models to study structure/biological activities relationships. Int J Mol Sci.

[9] Cavada BS, Osterne VJS, Lossio CF, Pinto-Junior VR, Oliveira MV, Silva MTL, Leal RB, Nascimento KS (2019). One century of ConA and 40 years of ConBr research: a structural review. Int J Biol Macromol.

[10] Shi Z, Li W, Tang Y, Cheng L (2017). A novel molecular model of plant lectin-induced programmed cell death in cancer. Biol Pharm Bull.

[11] Liu Z, Luo Y, Zhou TT, Zhang WZ (2013). Could plant lectins become promising anti-tumour drugs for causing autophagic cell death?. Cell Prolif.

[12] Estrada-Martínez LE, Moreno-Celis U, Cervantes-Jimenez R, Ferriz-Martínez RA, Blanco-Labra A, Garcia-Gasca T (2017). Plant lectins as medical tools against digestive system cancers. Int J Mol Sci.

[13] L-l Fu, Zhou C, Yao S, Yu J, Liu B, Bao J (2011). Plant lectins: targeting programmed cell death pathways as antitumor agents. Int J Biochem Cell Biol.

[14] Yau T, Dan X, Ng CCW, Ng TB (2015). Lectins with potential for anti-cancer therapy. Molecules.

[15] Bazeed M, Becht E, Scharfe T, Schmidt J, Jacobi G, Thüjroff J (1988). Effect of lectins on KK-47 bladder cancer cell line. Urology.

[16] Yu M, Sato H, Seiki M, Spiegel S, Thompson EW (1998). Elevated cyclic AMP suppresses ConA-induced MT1-MMP expression in MDA-MB-231 human breast cancer cells. Clin Exp Metas.

[17] Nanni SB, Pratt J, Beauchemin D, Haidara K, Annabi B (2016). Impact of concanavalin-A-mediated cytoskeleton disruption on low-density lipoprotein receptor-related protein-1 internalization and cell surface expression in glioblastomas. Biomark Cancer.

[18] Liu B, Min M, Bao J-K (2009). Induction of apoptosis by Concanavalin A and its molecular mechanisms in cancer cells. Autophagy.

[19] Sato H, Takino T, Miyamori H (2005). Roles of membrane-type matrix metalloproteinase-1 in tumor invasion and metastasis. Cancer Sci.

[20] Sounni NE, Noël A (2005). Membrane type-matrix metalloproteinases and tumor progression. Biochimie.

[21] Yamamoto M, Mohanam S, Sawaya R, Fuller GN, Seiki M, Sato H, Gokaslan ZL, Liotta LA, Nicolson GL, Rao JS (1996). Differential expression of membrane-type matrix metalloproteinase and its correlation with gelatinase A activation in human malignant brain tumors in vivo and in vitro. Cancer Res.

[22] Gálvez BG, Matías-Román S, Yáñez-Mó M, Vicente-Manzanares M, Sánchez-Madrid F, Arroyo AG (2004). Caveolae are a novel pathway for membrane-type 1 matrix metalloproteinase traffic in human endothelial cells. Mol Biol Cell.

[23] Thant AA, Serbulea M, Kikkawa F, Liu E, Tomoda Y, Hamaguchi M (1997). c-Ras is required for the activation of the matrix metalloproteinases by concanavalin A in 3Y1 cells. FEBS Lett.

[24] Gingras D, Pagé M, Annabi B, Bëliveau R (2000). Rapid activation of matrix metalloproteinase-2 by glioma cells occurs through a posttranslational MT1-MMP-dependent mechanism. Biochim Biophys Acta (BBA) Mol Cell Res.

[25] Kim S, Huang W, Mottillo EP, Sohail A, Ham Y-A, Conley-LaComb MK, Kim CJ, Tzivion G, Kim H-RC, Wang S (2010). Posttranslational regulation of membrane type 1-matrix metalloproteinase (MT1-MMP) in mouse PTEN null prostate cancer cells: enhanced surface expression and differential O-glycosylation of MT1-MMP. Biochim Biophys Acta (BBA) Mol Cell Res.

[26] Yu M, Sato H, Seiki M, Spiegel S, Thompson EW (1997). Calcium influx inhibits MT1-MMP processing and blocks MMP-2 activation. FEBS Lett.

[27] Yu M, Bowden ET, Sitlani J, Sato H, Seiki M, Mueller SC, Thompson EW (1997). Tyrosine phosphorylation mediates ConA-induced membrane type 1-matrix metalloproteinase expression and matrix metalloproteinase-2 activation in MDA-MB-231 human breast carcinoma cells. Cancer Res.

[28] Domoto T, Takino T, Guo L, Sato H (2012). Cleavage of hepatocyte growth factor activator inhibitor-1 by membrane-type MMP-1 activates matriptase. Cancer Sci.

[29] Akla N, Pratt J, Annabi B (2012). Concanavalin-A triggers inflammatory response through JAK/STAT3 signalling and modulates MT1-MMP regulation of COX-2 in mesenchymal stromal cells. Exp Cell Res.

[30] Zhao R, Guerrah A, Tang H, Zhao ZJ (2002). Cell surface glycoprotein PZR is a major mediator of concanavalin A-induced cell signaling. J Biol Chem.

[31] Sulová Z, Ditte P, Kurucová T, Poláková E, Rogozánová K, Gibalová L, Šereš M, Škvarková L, Sedlák J, Pastorek J (2010). The presence of P-glycoprotein in L1210 cells directly induces down-regulation of cell surface saccharide targets of concanavalin A. Anticancer Res.

[32] Matsuo T, Hazeki K, Hazeki O, Katada T, Ui M (1996). Activation of phosphatidylinositol 3-kinase by concanavalin A through dual signaling pathways, G-protein-coupled and phosphotyrosine-related, and an essential role of the G-protein-coupled signals for the lectin-induced respiratory burst in human monocytic THP-1 cells. Biochem J.

[33] Fan X, Men R, Huang C, Shen M, Wang T, Ghnewa Y, Ma Y, Ye T, Yang L (2020). Critical roles of conventional dendritic cells in autoimmune hepatitis via autophagy regulation. Cell Death Dis.

[34] Torti M, Ramaschi G, Sinigaglia F, Balduini C (1995). Dual mechanism of protein-tyrosine phosphorylation in concanavalin A-stimulated platelets. J Cell Biochem.

[35] Vinnakota K, Hu F, Ku M-C, Georgieva PB, Szulzewsky F, Pohlmann A, Waiczies S, Waiczies H, Niendorf T, Lehnardt S (2013). Toll-like receptor 2 mediates microglia/brain macrophage MT1-MMP expression and glioma expansion. Neuro Oncol.

[36] Zgheib A, Lamy S, Annabi B (2013). Epigallocatechin gallate targeting of membrane type 1 matrix metalloproteinase-mediated Src and Janus kinase/signal transducers and activators of transcription 3 signaling inhibits transcription of colony-stimulating factors 2 and 3 in mesenchymal stromal cells. J Biol Chem.

[37] Zhou M, Zhu X, Ye S, Zhou B (2014). Blocking TLR2 in vivo attenuates experimental hepatitis induced by concanavalin A in mice. Int Immunopharmacol.

[38] Ojiro K, Ebinuma H, Nakamoto N, Wakabayashi K, Mikami Y, Ono Y, Po-Sung C, Usui S, Umeda R, Takaishi H (2010). MyD88-dependent pathway accelerates the liver damage of Concanavalin A-induced hepatitis. Biochem Biophys Res Commun.

[39] Wang K, Wang X, Hou Y, Zhou H, Mai K, He G (2019). Apoptosis of cancer cells is triggered by selective crosslinking and inhibition of receptor tyrosine kinases. Commun Biol.

[40] Nascimento APM, Wolin IA, Welter PG, Heinrich IA, Zanotto-Filho A, Osterne VJ, Lossio CF, Silva MT, Nascimento KS, Cavada BS (2019). Lectin from Dioclea violacea induces autophagy in U87 glioma cells. Int J Biol Macromol.

[41] Belkaid A, Fortier S, Cao J, Annabi B (2007). Necrosis induction in glioblastoma cells reveals a new “bioswitch” function for the MT1-MMP/G6PT signaling axis in proMMP-2 activation versus cell death decision. Neoplasia.

[42] Roy B, Pattanaik AK, Das J, Bhutia SK, Behera B, Singh P, Maiti TK (2014). Role of PI3K/Akt/mTOR and MEK/ERK pathway in Concanavalin A induced autophagy in HeLa cells. Chem Biol Interact.

[43] Amin AR, Thakur VS, Gupta K, Jackson MW, Harada H, Agarwal MK, Shin DM, Wald DN, Agarwal ML (2010). Restoration of p53 functions protects cells from Concanavalin A-induced apoptosis. Mol Cancer Ther.

[44] Shi Z, Chen J, Li C, An N, Wang Z, Yang S-I, Huang K, Bao J (2014). Antitumor effects of concanavalin A and Sophora flavescens lectin in vitro and in vivo. Acta Pharmacol Sin.

[45] Amin AR, Paul RK, Thakur VS, Agarwal ML (2007). A novel role for p73 in the regulation of Akt-Foxo1a-Bim signaling and apoptosis induced by the plant lectin Concanavalin A. Cancer Res.

[46] Liu B, Li C, Bian H, Min M, Chen L, Bao J (2009). Antiproliferative activity and apoptosis-inducing mechanism of Concanavalin A on human melanoma A375 cells. Arch Biochem Biophys.

[47] Faheina-Martins GV, da Silveira AL, Cavalcanti BC, Ramos MV, Moraes MO, Pessoa C, Araújo DA (2012). Antiproliferative effects of lectins from Canavalia ensiformis and Canavalia brasiliensis in human leukemia cell lines. Toxicol In Vitro.

[48] Chang CP, Yang MC, Liu HS, Lin YS, Lei HY (2007). Concanavalin A induces autophagy in hepatoma cells and has a therapeutic effect in a murine in situ hepatoma model. Hepatology.

[49] Pratt J, Roy R, Annabi B (2012). Concanavalin-A-induced autophagy biomarkers requires membrane type-1 matrix metalloproteinase intracellular signaling in glioblastoma cells. Glycobiology.

[50] Pratt J, Annabi B (2014). Induction of autophagy biomarker BNIP3 requires a JAK2/STAT3 and MT1-MMP signaling interplay in Concanavalin-A-activated U87 glioblastoma cells. Cell Signal.

[51] Desjarlais M, Pratt J, Lounis A, Mounier C, Haidara K, Annabi B (2014). Tetracycline derivative minocycline inhibits autophagy and inflammation in concanavalin-A-activated human hepatoma cells. Gene Regul Syst Biol.

[52] Annabi B, Laflamme C, Sina A, Lachambre M-P, Béliveau R (2009). A MT1-MMP/NF-κB signaling axis as a checkpoint controller of COX-2 expression in CD133 (+) U87 glioblastoma cells. J Neuroinflamm.

[53] Sina A, Proulx-Bonneau S, Roy A, Poliquin L, Cao J, Annabi B (2010). The lectin concanavalin-A signals MT1-MMP catalytic independent induction of COX-2 through an IKKγ/NF-κB-dependent pathway. J Cell Commun Signal.

[54] Proulx-Bonneau S, Pratt J, Annabi B (2011). A role for MT1-MMP as a cell death sensor/effector through the regulation of endoplasmic reticulum stress in U87 glioblastoma cells. J Neurooncol.

[55] Li J-Z, Zhou X-X, Wu W-Y, Qiang H-F, Xiao G-S, Wang Y, Li G (2022). Concanavalin A promotes angiogenesis and proliferation in endothelial cells through the Akt/ERK/Cyclin D1 axis. Pharm Biol.

[56] Kulkarni G, McCulloch C (1995). Concanavalin A induced apoptosis in fibroblasts: the role of cell surface carbohydrates in lectin mediated cytotoxicity. J Cell Physiol.

[57] Biswas MHU, Hasegawa HH, Rahman MA, Huang P, Mon NN, Amin AR, Senga T, Kannagi R, Hamaguchi M (2006). SHP-2-Erk signaling regulates concanavalin A-dependent production of TIMP-2. Biochem Biophys Res Commun.

[58] Overall C, Sodek J (1990). Concanavalin A produces a matrix-degradative phenotype in human fibroblasts. Induction and endogenous activation of collagenase, 72-kDa gelatinase, and Pump-1 is accompanied by the suppression of the tissue inhibitor of matrix metalloproteinases. J Biol Chem.

[59] Amin AR, Oo ML, Senga T, Suzuki N, Feng G-S, Hamaguchi M (2003). SH2 domain containing protein tyrosine phosphatase 2 regulates concanavalin A-dependent secretion and activation of matrix metalloproteinase 2 via the extracellular signal-regulated kinase and p38 pathways. Cancer Res.

[60] Takino T, Miyamori H, Watanabe Y, Yoshioka K, Seiki M, Sato H (2004). Membrane type 1 matrix metalloproteinase regulates collagen-dependent mitogen-activated protein/extracellular signal-related kinase activation and cell migration. Cancer Res.

[61] Chang C-P, Cheng W-C, Lei H-Y (2005). A cellular ELISA to screen lectin-like compounds for cancer cell binding. Lett Drug Des Discov.

[62] Saito M, Takaku F, Hayashi M, Tanaka I, Abe Y, Nagai Y, Ishii S (1983). A role of valency of concanavalin A and its chemically modified derivatives in lymphocyte activation. Monovalent monomeric concanavalin A derivative can stimulate lymphocyte blastoid transformation. J Biol Chem.

[63] Ohta S, Inazu T, Taniguchi T, Nakagawara G (1992). YAMAMURA H: Protein-tyrosine phosphorylations induced by concanavalin A and N-formyl-methionyl-leucyl-phenylalanine in human neutrophils. Eur J Biochem.

[64] Simon-Molas H, Vallvé-Martínez X, Caldera-Quevedo I, Fontova P, Arnedo-Pac C, Vidal-Alabró A, Castaño E, Navarro-Sabaté À, Lloberas N, Bartrons R (2021). TP53-induced glycolysis and apoptosis regulator (TIGAR) is upregulated in lymphocytes stimulated with Concanavalin A. Int J Mol Sci.

[65] Simon-Molas H, Arnedo-Pac C, Fontova P, Vidal-Alabró A, Castaño E, Rodríguez-García A, Navarro-Sabaté À, Lloberas N, Manzano A, Bartrons R (2018). PI3K–Akt signaling controls PFKFB3 expression during human T-lymphocyte activation. Mol Cell Biochem.

[66] Erhardt A, Biburger M, Papadopoulos T, Tiegs G (2007). IL-10, regulatory T cells, and Kupffer cells mediate tolerance in concanavalin A-induced liver injury in mice. Hepatology.

[67] Hong F, Jaruga B, Kim WH, Radaeva S, El-Assal ON, Tian Z, Nguyen V-A, Gao B (2002). Opposing roles of STAT1 and STAT3 in T cell-mediated hepatitis: regulation by SOCS. J Clin Investig.

[68] Miyagi T, Takehara T, Tatsumi T, Suzuki T, Jinushi M, Kanazawa Y, Hiramatsu N, Kanto T, Tsuji S, Hori M (2004). Concanavalin a injection activates intrahepatic innate immune cells to provoke an antitumor effect in murine liver. Hepatology.

[69] Kato M, Ikeda N, Matsushita E, Kaneko S, Kobayashi K (2001). Involvement of IL-10, an anti-inflammatory cytokine in murine liver injury induced by Concanavalin A. Hepatol Res.

[70] Mertz PM, DeWitt DL, Stetler-Stevenson WG, Wahl LM (1994). Interleukin 10 suppression of monocyte prostaglandin H synthase-2. Mechanism of inhibition of prostaglandin-dependent matrix metalloproteinase production. J Biol Chem.

[71] Chen W-Y, Cheng Y, Lei H, Chang C, Wang C, Chang M-S (2005). IL-24 inhibits the growth of hepatoma cells in vivo. Genes Immun.

[72] Zheng C, Yin S, Yang Y, Yu Y, Xie X (2018). CD24 aggravates acute liver injury in autoimmune hepatitis by promoting IFN-γ production by CD4+ T cells. Cell Mol Immunol.

[73] KanekoY HM (2000). Augmentation of Va14 NKT cell-mediated cytotoxicity by interleukin 4 in an autocrine mechanism resulting in the development of concanavalin A-induced hepatitis. J Exp Med.

[74] Xu X, Wei H, Dong Z, Chen Y, Tian Z (2006). The differential effects of low dose and high dose concanavalin A on cytokine profile and their importance in liver injury. Inflamm Res.

[75] Wang Y, Feng D, Wang H, Xu M-J, Park O, Li Y, Gao B (2014). STAT4 knockout mice are more susceptible to concanavalin A-induced T-cell hepatitis. Am J Pathol.

[76] Morinobu A, Gadina M, Strober W, Visconti R, Fornace A, Montagna C, Feldman GM, Nishikomori R, O'Shea JJ (2002). STAT4 serine phosphorylation is critical for IL-12-induced IFN-γ production but not for cell proliferation. Proc Natl Acad Sci.

[77] Radaeva S, Sun R, Pan H, Hong F, Gao B (2004). Interleukin 22 (IL-22) plays a protective role in T cell-mediated murine hepatitis: IL-22 is a survival factor for hepatocytes via STAT3 activation. Hepatology.

[78] Ye F, Yan S, Xu L, Jiang Z, Liu N, Xiong S, Wang Y, Chu Y (2009). Tr1 regulatory T cells induced by ConA pretreatment prevent mice from ConA-induced hepatitis. Immunol Lett.

[79] Caterino S. Modulation of immune response: Parp-1 plays opposite roles in Th2 and regulatory T cell differentiation.

[80] Fukaura H, Kent SC, Pietrusewicz MJ, Khoury SJ, Weiner HL, Hafler DA (1996). Induction of circulating myelin basic protein and proteolipid protein-specific transforming growth factor-beta1-secreting Th3 T cells by oral administration of myelin in multiple sclerosis patients. J Clin Investig.

[81] Battaglia M, Gregori S, Bacchetta R, Roncarolo M-G (2006). Tr1 cells: from discovery to their clinical application. Semin Immunol.

[82] Levings MK, Roncarolo M-G (2000). T-regulatory 1 cells: a novel subset of CD4+ T cells with immunoregulatory properties. J Allergy Clin Immunol.

[83] Wang H-X, Liu M, Weng S-Y, Li J-J, Xie C, He H-L, Guan W, Yuan Y-S, Gao J (2012). Immune mechanisms of Concanavalin A model of autoimmune hepatitis. World J Gastroenterol WJG.

[84] Danielsson Å, Prytz H (1994). Oral budesonide for treatment of autoimmune chronic active hepatitis. Aliment Pharmacol Ther.

[85] Chen L, Xie X-J, Ye Y-F, Zhou L, Xie H-Y, Xie Q-F, Tian J, Zheng S-S (2011). Kupffer cells contribute to concanavalin A-induced hepatic injury through a Th1 but not Th17 type response-dependent pathway in mice. Hepatobiliary Pancreat Dis Int.

[86] Sahoo SS, Majhi RK, Tiwari A, Acharya T, Kumar PS, Saha S, Kumar A, Goswami C, Chattopadhyay S: Transient receptor potential ankyrin1 channel is endogenously expressed in T cells and is involved in immune functions. *Bioscience reports* 2019, 39:BSR20191437. 10.1042/BSR20191437 PMC6753326 31488616

[87] Andrade JL, Arruda S, Barbosa T, Paim L, Ramos MV, Cavada BS, Barral-Netto M (1999). Lectin-induced nitric oxide production. Cell Immunol.

[88] Sorg C (1980). Characterization of murine macrophage migration inhibitory activities (MIF) released by concanavalin A stimulated thymus or spleen cells. Mol Immunol.

[89] Klinkert W, Sorg C (1980). Characterization of four lymphocyte activation products of guinea pig and their association with macrophage migration inhibitory activity (MIF). Mol Immunol.

[90] Lai Y, Chuang Y, Chang C, Yeh T (2015). Macrophage migration inhibitory factor has a permissive role in concanavalin A-induced cell death of human hepatoma cells through autophagy. Cell Death Dis.

[91] Nakajima H, Takagi H, Horiguchi N, Toyoda M, Kanda D, Otsuka T, Emoto Y, Emoto M, Mori M (2006). Lack of macrophage migration inhibitory factor protects mice against concanavalin A-induced liver injury. Liver Int.

[92] Li C-Y, Xu H-L, Liu B, Bao J-K (2010). Concanavalin A, from an old protein to novel candidate anti-neoplastic drug. Curr Mol Pharmacol.

[93] Li W-w, Yu J-y, Xu H-l, Bao J-k (2011). Concanavalin A: a potential anti-neoplastic agent targeting apoptosis, autophagy and anti-angiogenesis for cancer therapeutics. Biochem Biophys Res Commun.

[94] Pang Q, Jin H, Wang Y, Dai M, Liu S, Tan Y, Liu H, Lu Z (2021). Depletion of serotonin relieves concanavalin A-induced liver fibrosis in mice by inhibiting inflammation, oxidative stress, and TGF-β1/Smads signaling pathway. Toxicol Lett.

